# Adaptive fluorescence lifetime imaging with per-pixel signal optimization and flexible scanning

**DOI:** 10.1364/BOE.566518

**Published:** 2025-09-23

**Authors:** Siyuan Xie, Gareth O. S. Williams, Ahsan R. Akram, Ahmet T. Erdogan, James R. Hopgood

**Affiliations:** 1 Centre for Inflammation Research, Institute for Regeneration and Repair, The University of Edinburgh, Edinburgh, UK; 2 School of Engineering, Institute for Integrated Micro and Nano Systems, The University of Edinburgh, Edinburgh, UK; 3 School of Engineering, Institute for Imaging, Data, and Communications, The University of Edinburgh, Edinburgh, UK

## Abstract

Advances in time-resolved fluorescence lifetime imaging microscopy (FLIM) have significantly enhanced biological imaging compared to steady-state techniques alone. The primary goal of modern FLIM is to acquire high-resolution fluorescence lifetime profiles with a high signal-to-noise ratio (SNR) from heterogeneous samples at high speeds, posing challenges in balancing imaging speed, signal strength, and sample integrity. In this study, we present fluorescence lifetime intensity-inverted imaging microscopy (FLI^3^M), an adaptive imaging technique based on confocal laser scanning microscopy (CLSM) that dynamically adjusts pixel dwell times using *a priori* intensity information from a pre-scan and supports flexible scanning patterns. This approach achieves uniform SNR imaging by either providing up to an eight-fold signal enhancement without increasing imaging time or reducing imaging time without compromising SNR. We demonstrate the potential of this technique through imaging studies of biological samples, including *Convallaria majalis* and human lung tissue. The results show a 56% average improvement in fluorescence lifetime estimation reliability in low-SNR regions and an increase in imaging speed ranging from 27% to 53% across various samples. This enables detailed resolution of optical fingerprints in complex biological environments that are challenging for conventional imaging. Collectively, these results establish our adaptive FLIM system as a powerful tool for high-performance cellular imaging, FLIM-guided diagnostics, and a wide range of biomedical applications.

## 1. Introduction

Fluorescence lifetime, defined as the average time a fluorophore remains in its excited state, has become an essential tool for probing microenvironments and intermolecular signal exchanges in complex biological systems due to its relative independence from fluorophore concentration [1,2]. Time-resolved fluorescence lifetime imaging microscopy (FLIM) provides fluorescence lifetime information on the spatial distribution of fluorophores, further enhancing its utility in biological research and clinical diagnostics [3–5]. However, most FLIM experiments, such as those monitoring biological processes, face two significant constraints: they are either time sensitive due to the need to monitor dynamic changes within specific times, or limited by a photon budget in order to preserve sample integrity from photodamage [6,7]. Biologists often select photon-stable fluorophores to label samples; however, this approach requires additional sample preparation for *in vivo* labeling and is not possible for imaging based on autofluorescence [8]. To address low-light conditions, imaging scientists have developed specific lifetime determination algorithms (LDAs) to improve lifetime estimation accuracy [9]. Nevertheless, the accuracy is inherently limited by the signal-to-noise ratio (SNR), which, in photon-counting FLIM, is proportional to the square root of the detected photons ( $\sqrt{N}$ ) [10].

In conventional confocal laser scanning microscopy (CLSM), scanning is performed row by row, with each scan point assigned the same dwell time (exposure time) while the laser power remains constant across all points (image pixels) [11,12]. A common approach to enhance the SNR therefore is to globally increase either the excitation power or the pixel exposure time until weakly fluorescent areas produce signals strong enough to resolve structures and determine lifetimes. However, this method has significant drawbacks. In fluorophore-dense regions, the prolonged exposure required for low-light areas results in an excessively high local SNR, unnecessarily increasing the risk of photodamage. In regions without fluorophores, increasing the light dose does not improve SNR due to the absence of signal; instead, it increases dark counts (noise). Moreover, increasing the exposure time in scanning-based imaging systems can significantly prolong overall acquisition. Balancing these trade-offs while optimizing excitation parameters remains a major challenge in FLIM applications.

Adaptive imaging approaches provide additional strategies for improving SNR during acquisition. In complex biological environments, signal strength can vary by orders of magnitude over micrometer scales due to fluctuating expression levels and local concentrations [13]. As a result, biological samples are often spatially and temporally heterogeneous, meaning that illumination patterns designed to achieve a uniform SNR image are non-uniform in space and time [14]. A more efficient approach involves selectively adjusting the exposure time based on fluorescence intensity. Regions with fluorescence below a threshold (dominated by dark counts) can be skipped to save time without losing relevant information, while weakly fluorescent areas can receive longer exposure for signal enhancement. Conversely, strongly fluorescent regions, including saturated areas, can have reduced exposure to minimize the risk of photodamage with minimal signal loss. This strategy aligns with the SNR behavior in photon-counting FLIM, where additional photon counts yield greater relative SNR improvements at low photon levels than at high photon levels.

Previous studies have demonstrated adaptive imaging strategies in point-scanning confocal, multiphoton, and stimulated emission depletion (STED) microscopy through feedback mechanisms linking the sample and hardware, as summarized in Table 1 [15–21]. Point-based feedback typically uses electronic circuits to modulate the light dose, such as by modulating an acousto-optic device, based on real-time signals from the detector. It dynamically calculates the signal for image formation on the fly [15,16]. This approach enables point-by-point illumination control as the scanning beam moves across the sample and is typically achieved on a microsecond timescale. However, this method is mainly restricted to conventional fluorescence intensity imaging, where the readout captures discrete spectral bands without temporal resolution. In contrast, multiplexed FLIM, which requires both spectral and temporal data acquisition, may introduce significant feedback delays due to the increased data processing and readout load [22]. An alternative approach is frame-based feedback, in which a pre-scan is performed by raster scanning the sample, followed by a second imaging pass with an adapted illumination pattern based on the pre-scan data [17]. However, the added time from the pre-scan must be considered, as it may offset the benefits of adaptive imaging and raise questions about its overall efficiency compared to a single long-exposure raster scan.

**Table 1.** Summary of adaptive illumination control techniques in microscopy

Microscopy Modality	Feedback Type	Illumination Control	Objective	Reference
Confocal laser scanning microscopy	Point-based	Acoustic-optical modulator	Reduces photobleaching and phototoxicity	[15]
Two-photon laser scanning microscopy	Point-based	Electro-optic modulator	Enhances sensitivity and dynamic range	[16]
Multiphoton microscopy	Frame-based	Fiber electro-optic modulator	Increases speed and sensitivity	[17]
Structured illumination microscopy	Frame-based	Digital micromirror device	Reduces photobleaching	[18]
Stimulated emission depletion microscopy	Frame-based	Electro-optic scanners	Increases resolution and SNR	[19]
Optical linear fluorescence transitions nanoscopy	Point-based	Field-programmable gate array (FPGA)-controlled modulation	Increases speed and reduces light exposure	[20]
Stimulated emission depletion nanoscopy	Point-based	Electro-optic deflectors and galvanometer scanners	Increases speed and reduces phototoxicity	[21]
Confocal laser scanning microscopy	Frame-based	Non-resonant galvanometer scanners	Optimizes SNR and increases speed	This paper

Here, we introduce Fluorescence Lifetime Intensity-Inverted Imaging Microscopy (FLI^3^M), an advanced adaptive imaging technique developed using an inverted confocal microscope. This method enables full region-of-interest (ROI) scanning with precise SNR correction, providing key advantages across a range of imaging modalities. First, FLI^3^M controls light dose by adjusting pixel dwell times instead of modulating excitation power. Each pixel’s dwell time is synchronized with the movement of non-resonant galvanometer (galvo) scanners, so that a reduction in dwell time directly decreases the overall frame acquisition time. This allows for per-pixel SNR corrections by assigning longer dwell times to weakly fluorescent regions and shorter dwell times to fluorescence-dense regions. This approach achieves uniform SNR across the image while maintaining imaging speeds comparable to conventional raster scanning. Second, the feedback loop operates on a per-frame basis using a pre-scan to capture not only intensity profiles but also photon timing data. This information can be fully utilized during adaptive imaging, leading to enhanced fluorescence lifetime estimation while maximizing time efficiency and preventing information loss. In this study, we examine the depth of fluorescence lifetime information acquired using FLI^3^M on biological samples, including human lung tissue for histological analysis. The results demonstrate the potential of our adaptive imaging system to resolve complete optical fingerprints in complex biological structures that are challenging for conventional imaging techniques.

## 2. Methods

### 2.1. Optical system

**Fig. 1.**
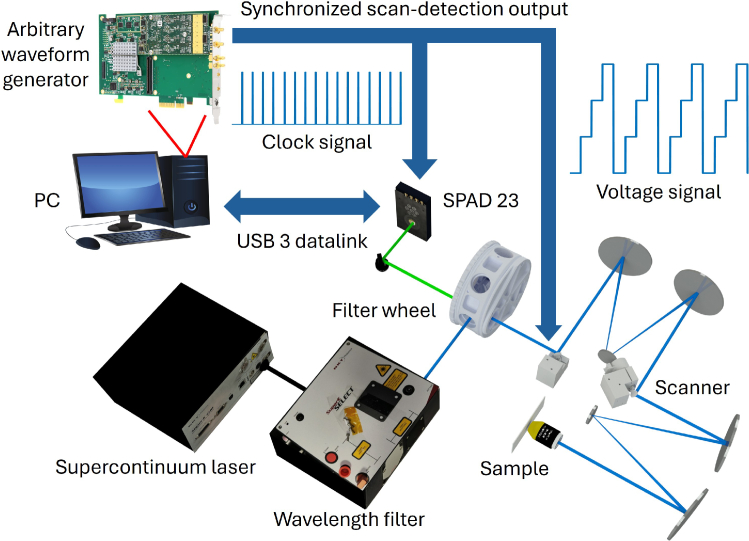
Schematic of the FLIM system setup (not to scale). The system directs up to eight filtered laser wavelengths to a filter wheel, where a dichroic mirror reflects the beam into the scanning optics. The excitation and back-fluorescence light paths are shown in blue and green, respectively. This optical layout is adapted from a previously published design featuring a fully reflective optical path to minimize chromatic aberration [26].

Figure 1 shows the complete layout of the system, which is based on a time-correlated single-photon counting (TCSPC) setup. The detector features an array of 23 single-photon avalanche diodes (SPADs), each paired with an individual time-to-digital converter (TDC) (Pi Imaging, SPAD 23, 10 ps temporal resolution) for time-resolved fluorescence lifetime imaging [23,24]. The SPADs array enables higher photon throughput of up to 90 million counts per second, effectively mitigating common challenges in FLIM experiments, such as the "pile-up" effect [25]. The super-continuum laser (NKT Evo HP, pulse width of less than 100 ps, pulse repetition rate 20 MHz) is filtered using an acousto-optic tunable filter (NKT Super-K Select), producing up to eight spectral lines between 400 and 700 nm, each with a bandwidth of approximately 2 nm and an average power of around 250 μ W. The collimated beam is directed onto a two-dimensional, non-resonant galvanometer scanner (Thorlabs GVS202) for beam scanning. The beam position is controlled by two-channel analog voltages generated by an arbitrary waveform generator (AWG) (Spectrum Instrumentation GmbH, M2p.6541-x4), with a scaling factor of 1 volt per degree. This scanning system produces an XY field of view up to 450 μ m × 450 μ m ( ± 3 degrees) using a primary objective (Olympus 10 × 0.3 UplanFL air objective). The fluorescence signal is de-scanned through the same optical path and separated from the excitation beam by a dichroic filter (Semrock 488 nm BrightLine long-pass) mounted in a five-slot filter wheel (Thorlabs CSE2000W), enabling quick changes to the cut-on wavelength. The filtered light is collected by the SPAD 23 in TCSPC mode with a temporal resolution of 10 ps and transmitted to the PC via USB 3.0 data link. To ensure synchronized scanning and detection, digital dwell and line clocks are embedded in the scan pattern stored in the AWG and simultaneously sent to the SPAD 23 during scanning.

### 2.2. FLI^3^M

**Fig. 2.**
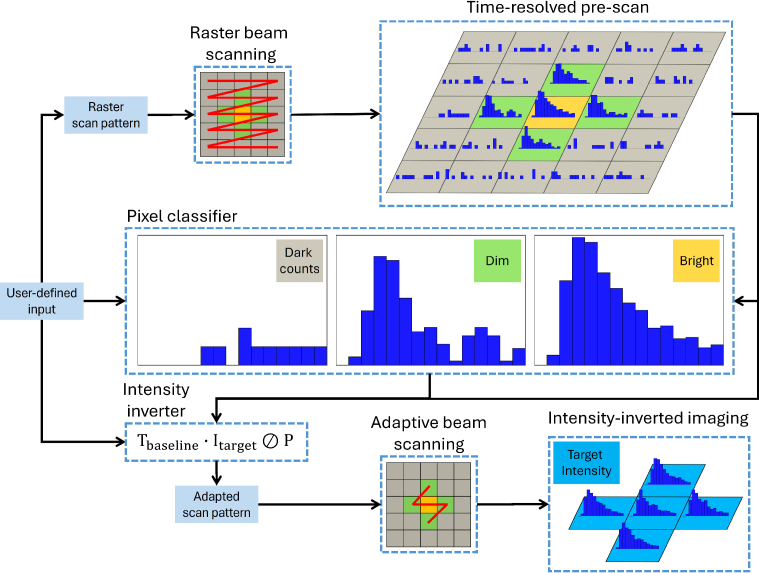
Flowchart of FLI^3^M implementation. The imaging process is illustrated using a 5×5 artificial image with centrally simulated fluorescence decays. User-defined inputs include: 1) Pre-scan parameters: pre-scan exposure time $T_{\text{prescan}}$ and scanner step size $V_{s}$ , which determine the pre-scan image formation. 2) ROI determination: the sensor’s dark count rate (DCR) and the intensity target $I_{\text{target}}$ , which are used by the pixel classifier to identify the ROI. 3) Adaptive exposure: baseline exposure time $T_{\text{baseline}}$ , which is adjusted by the intensity inverter to generate the adaptive exposure map. The symbol ⊘ denotes element-wise Hadamard division.

The core principle of FLI^3^M is to dynamically adjust exposure times for each image pixel based on the ratio of a predefined intensity target to the original intensities recorded during a pre-scan. Dim regions receive longer exposure times, while bright regions have reduced exposure times. By setting the exposure to 0 for certain pixels, they can be skipped entirely to reduce the overall scanning time. This non-uniform scan pattern optimizes both acquisition time and image quality. Figure 2 shows the implementation pipeline for FLI^3^M using a 5 × 5 artificial image. The implementation of FLI^3^M proceeds through the following steps:

Step 1: Sample pre-scanning. We input the predefined imaging parameters, including the image size M×N , pre-scan exposure time $T_{\text{prescan}}$ , and voltage step $V_{\text{s}}$ (which, together with the image size, determines the total field of view), into the imaging system to perform a raster scan. The detected photon timing data is then transferred to the PC for post-processing.

Step 2: Pixel classification. The photon counts from the pre-scan image are first evaluated against the sensor dark count rate (DCR) and adaptive thresholds determined by Otsu’s method to segment pixels into ROI and non-ROI groups [27]. Within the ROI, pixels are further classified into bright and dim subgroups based on one or multiple intensity thresholds, $I_{\text{target}}$ . These thresholds can be adjusted to achieve various objectives, such as preserving sample viability while optimizing fluorescence output, meeting the minimum SNR requirements of a lifetime estimator, or achieving a specific SNR level.

Step 3: Intensity inversion. The ratio of the target intensity to the pre-scan pixel intensity is calculated. Given that fluorescence intensity scales linearly with exposure time before saturation, changes in exposure time lead to proportional changes in photon counts. A ratio greater than 1 indicates an increase in exposure, whereas a ratio less than 1 indicates a decrease. By multiplying this ratio by the baseline exposure time $T_{\text{baseline}}$ , we obtain the adaptive exposure map. Steps 2 and 3 can be mathematically defined as follows:

(1) $$E(m,n)=\left\{ \begin{array}{ll} T_{\text{baseline}}\frac{I_{\text{target}}}{P(m,n\;|\;T_{\text{prescan}})} & \text{if }P(m,n\;|\;T_{\text{prescan}})>\alpha (m,n),\\ 0 & \text{otherwise}, \end{array}\right.$$

and

(2) $$\alpha (m,n)=\max(\text{DCR},O_{\text{thresh}}(m,n)).$$

where E(m,n) is the adaptive exposure map at pixel (m,n) , and $T_{\text{baseline}}$ is the baseline exposure time for adaptive imaging. $P(m,n\;|\;T_{\text{prescan}})$ denotes the photon counts recorded at pixel (m,n) during the pre-scan acquisition with exposure time $T_{\text{prescan}}$ . Note that the choice of $T_{\text{baseline}}$ and $T_{\text{prescan}}$ is application-dependent. Specifically, a shorter $T_{\text{prescan}}$ enables faster pre-scanning to capture the relative intensity distribution without resolving fine lifetime decays, whereas a longer $T_{\text{baseline}}$ is beneficial for high-precision lifetime imaging. Since only relative signal strength is required during pre-scanning, the exact value of $T_{\text{prescan}}$ is not critical. Provided that sufficient photons are collected to distinguish fluorophore signals from background noise, the adaptive performance remains robust without the need for precise tuning. This tolerance reduces the need for re-optimization of $T_{\text{prescan}}$ across different samples or experimental sessions. In this study, we set $T_{\text{baseline}}=T_{\text{prescan}}$ for consistency. Here, α(m,n) is a function that returns the larger value between the sensor’s dark count rate (DCR) and the intensity threshold $O_{\text{thresh}}(m,n)$ at pixel (m,n) . The threshold $O_{\text{thresh}}(m,n)$ is calculated using an adaptive version of Otsu’s method.

Step 4: Coordinate-to-voltage mapping. The generated exposure map is then converted into a voltage waveform for uploading to the arbitrary waveform generator (AWG). The AWG operates by outputting analog voltages at a configurable but constant sampling rate. By controlling the number of samples held at the same voltage, each pixel’s exposure time can be accurately controlled. To enable flexible scanning paths, the voltage sequences are arranged as:

(3) $${\vec{V}}_{x}(m,n)=\left\{ \begin{array}{l} nV_{s}{\vec{1}}_{1\times fE(m,n)}\\ \;\text{if }E(m,n)\neq 0,E(m-1,n-1)\neq 0,\\ nV_{s}{\vec{1}}_{1\times \left(f+\beta \max(|m-m_{1}|,|n-n_{1}|)\right)E(m,n)}\\ \;\text{if }E(m,n)\neq 0,E(m-1,n-1)=0,\\ \emptyset \\ \;\text{if }E(m,n)=0. \end{array}\right.$$

(4) $${\vec{V}}_{y}(m,n)=\left\{ \begin{array}{l} mV_{s}{\vec{1}}_{1\times fE(m,n)}\\ \;\text{if }E(m,n)\neq 0,E(m-1,n-1)\neq 0,\\ mV_{s}{\vec{1}}_{1\times \left(f+\beta \max(|m-m_{1}|,|n-n_{1}|)\right)E(m,n)}\\ \;\text{if }E(m,n)\neq 0,E(m-1,n-1)=0,\\ \emptyset \\ \;\text{if }E(m,n)=0. \end{array}\right.$$

where ${\vec{V}}_{x}(m,n)$ and ${\vec{V}}_{y}(m,n)$ are the voltage sequences at coordinate (m,n) for scanning in the X and Y directions, respectively. $\left(m_{1},n_{1}\right)$ denotes the last coordinate where $E(m_{1},n_{1})\neq \;0$ , f is the AWG sampling rate, and β is the settling coefficient. In this process, a predefined number of samples is assigned to each pixel when its left adjacent pixel is also a ROI pixel. If two ROI pixels do not share a border, an additional number of settling samples is added to the voltage sequences to account for the scanners’ travel time. This adjustment is defined as the largest axial distance between the two pixels. Empirical testing based on the measured step response of the galvanometer scanner indicated that setting β=0.02f provides an effective balance between compensating for scanner lag and maintaining efficient imaging speed. The corresponding error estimation is presented in Supplementary Section 2.A. (see Supplement 1). To further refine the scanning trajectory, a 5-point moving average filter is applied to the generated waveform to smooth out abrupt changes in scanner speed. This smoothing introduces negligible impact on scanning accuracy, as only 6 out of 1000 samples per image pixel are affected at a 10 MS/s sampling rate. Steps 2 to 4 are implemented in MATLAB and are summarized in two algorithms provided in Supplementary Algorithms S1 and S2 (see Supplement 1).

Step 5: Uploading the voltage sequences $V_{x}$ and $V_{y}$ to the AWG.

To maximize pre-processing speed, the ROI in this study was determined based on dark counts combined with adaptive thresholding using Otsu’s method. More advanced, learning-assisted techniques for biomedical image segmentation have been extensively covered in the literature [28–30]. The imaging time is determined by the total distance traveled by the scanner. While optimizing the scanning path could further reduce imaging time, this approach is computationally intensive, as identifying the shortest path across a network of tens of thousands of pixels introduces a noticeable delay between the pre-scan and the subsequent adaptive scan. In our implementation, the pre-scan acquisition requires approximately 7 seconds for a 256×256 image at 100 μ s exposure time, ensuring minimal image distortion due to the inertia of the scanners (i.e., the scanner cannot instantly rotate to a new angle). Notably, the pre-scan is time-resolved, allowing photon timing data to be reused during the subsequent adaptive lifetime imaging without introducing any time redundancy. Depending on the imaging content, the exposure map can be optimized either to maintain the same SNR with faster imaging speeds, or to maintain the same acquisition time while enhancing signal strength. This dual-mode imaging capability is demonstrated in the Results section.

### 2.3. Ethical approval and tissue preparation

Tissue used in this study was obtained with ethical approval from the NHS Lothian REC and facilitated by the NHS Lothian SAHSC Bioresource (REC No: 15/ES/0094). Informed consent was obtained from all patients. Following surgical resection, a pathologist collected a sample that included the tumor margin extending into macroscopically non-cancerous tissue. The fresh tissue sample was then transported to the research institute for FLIM imaging (see Supplementary Fig. S4 in Supplement 1). Subsequently, the sample was fixed in paraformaldehyde, embedded in paraffin wax, and sectioned onto two 4 μ m slides. One slide was imaged unstained, while a consecutive section was stained with Hematoxylin and Eosin (H&E) for comparison. Histopathological evaluation subsequently confirmed the sample to be a pT2bN0M0 squamous cell carcinoma of the lung.

## 3. Results

### 3.1. FLI^3^M of Convallaria majalis

**Fig. 3.**
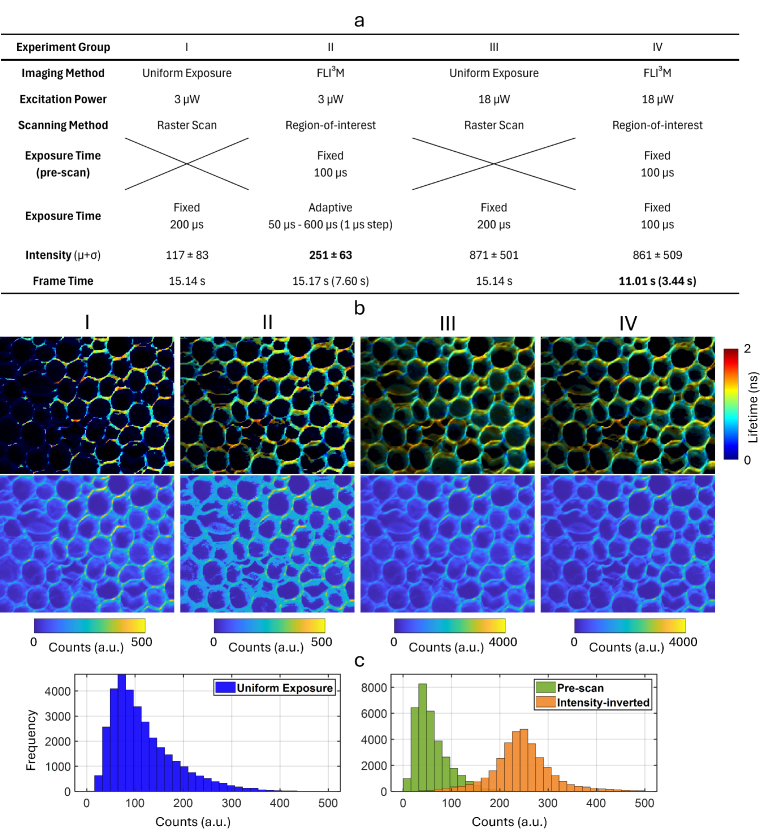
Imaging of *Convallaria majalis* using FLI^3^M and conventional uniform-exposure FLIM under two levels of excitation power. **a** Summary table of the experimental configurations for four imaging experiments conducted on the same sample region. Improvements achieved by FLI^3^M are highlighted in bold. For experiments using FLI^3^M, the reported frame times include both the pre-scan and subsequent adaptive imaging times (shown in parentheses). **b** Imaging results, including lifetime (top, with modulated transparency) and intensity (bottom), corresponding to the four experimental setups shown in panel a. **c** Intensity histograms from Experiments I (left) and II (right), showing both the pre-scan and the corresponding intensity-inverted photon distributions, compared to those obtained with the uniform exposure method.

Figure 3 compares FLI^3^M and conventional FLIM applied to sample *Convallaria majalis* stained with Safranin and Fast Green. In Fig. 3(a), we designed four experiments that use two levels of excitation power and imaging methods. Experiments I and II used a 3 μ W excitation power to simulate typical low-light imaging conditions, employing both the conventional method (uniform exposure with raster scanning) and FLI^3^M. Subsequently, the excitation power was increased to 18 μ W for experiments III and IV. The experiments were designed such that Experiments I and II had the same runtime, while Experiments III and IV achieved the same image quality (SNR). This setup demonstrates the dual-mode imaging capability of FLI^3^M, where SNR can be enhanced without increasing imaging time, or imaging time can be reduced without compromising SNR. To maximize imaging speed and preserve sample health, it is essential to use the lowest possible photon counts while maintaining sufficient reliability in lifetime estimation. The least-squares fitting has been the gold standard for lifetime analysis; however, it typically requires a moderate number of photons (>1000) [31]. The Centre-of-Mass method (CMM) was therefore chosen for lifetime determination because of its robustness in low-light conditions ( ∼ 250 photons) and ease of implementation [32]. The threshold for lifetime calculations was set at five times the peak intensity relative to background noise—defined as the peak intensity in the decay being at least five times the noise from dark counts and scattering within the optical system—corresponding to approximately 100 photons. Figure 3(b) shows the associated results for both intensity and lifetime. As observed in the lifetime image from experiment I, a significant number of pixels in the bottom left lack lifetime information, a common challenge in low-light imaging, with an average photon count per pixel of only 117. Additionally, large intensity fluctuations disrupt lifetime connectivity across the image, complicating both interpretation and detailed object-level analysis. To resolve this challenge, in Experiment II, a pre-scan was first performed (see Supplementary Fig. S2 in Supplement 1). We then employed the proposed FLI^3^M with both ROI scanning and intensity inversion enabled. Within the ROI, a target photon count of 250 was used to generate the exposure map. To avoid scanner latency and inconsistent exposure caused by extremely short or long exposure times, the exposure times were capped at 50 μ s and 600 μ s with 1 μ s steps. Image pixels outside of the ROI were assigned 0 μ s exposure, with the scanner moving directly to the next non-zero exposure pixel. The resulting intensity image shows that previously weakly fluorescent pixels are enhanced by assigning longer exposure times, increasing photon counts by an average of 117% without affecting the frame time. This effect is further illustrated in the corresponding intensity histograms for Experiments I and II (Fig. 3(c)). The ideal photon-count distribution for intensity-inverted imaging would resemble a Dirac-delta like impulse centered on a moderate to high count reflecting the desired SNR. However, as fluorescence emission is a stochastic process, in practice a Gaussian distribution centered at the position of the ideal impulse is to be expected. This is in contrast to the skewed distribution with high variance seen when a uniform exposure is applied. The histograms confirm that the photon count distributions in the FLI^3^M acquisitions closely follow the expected Gaussian-like behavior across spatial regions, exhibiting lower variability and reduced skewness compared to the uniform exposure method. This strategy provides an alternative to relying solely on increased excitation power to enhance the SNR, particularly in scenarios such as autofluorescence imaging, where fluorescence intensity is limited by dynamic quenching. In such cases, increasing the excitation power offers minimal improvement in image quality while substantially raising the risk of photodamage to the sample.

Next, we adjusted the excitation power from 3 μ W to 18 μ W to simulate imaging in a brighter scene for Experiments III and IV. At higher excitation power, Experiment III records an average photon count of 871, adequate for most non-fitting lifetime estimators, indicating that additional signal enhancement was unnecessary. Therefore, in Experiment IV, we only employed ROI scanning with a fixed exposure duration of 100 μ s, while all other parameters remained unchanged. Lifetime results show that both methods yield comparable images, despite FLI^3^M achieving a 27% faster imaging speed. While this comparison is based on single-frame imaging with reuse of pre-scan photon timings, the time savings become more pronounced in multi-frame acquisitions, where cumulative reductions in acquisition time can have a substantial impact.

### 3.2. FLI^3^M of unstained human lung tissue for histology

**Fig. 4.**
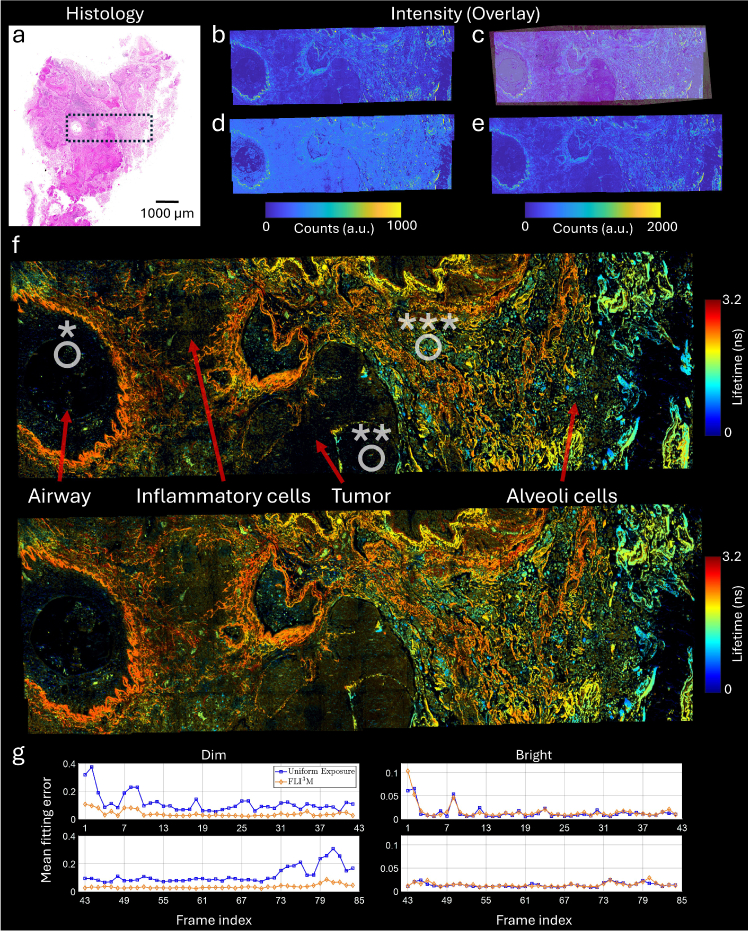
FLI^3^M of human lung tissue. **a** H&E stained histology image; dashed area approximates the unstained imaging region. **b** Pre-scan intensity image of the unstained slice, consisting of 6 rows of 14 (256 × 256 pixels) images, each with a 450 μ m × 450 μ m field of view. Imaging parameters: 100 μ s exposure, 735 μ W, 475 nm excitation, manually stitched with 20 μ m overlap, forming a 4 mm × 1.5 mm view. **c** Intensity image in panel b overlaid with the histology image. **d** Re-imaged intensity-inverted image based on pre-scan data, target 250 photons, adaptive exposure per pixel (50–800 μ s in 1 μ s steps). **e** Re-imaged uniform exposure image with 380 μ s exposure (24 s frame time). **f** Lifetime images for uniform exposure (top) and intensity-inverted (bottom) methods. A detailed analysis of three highlighted areas—an airway (*), a cancerous region (**), and a structurally complex region (***)—is provided in Fig. 5. **g** Mean lifetime fitting error (normalized) in dim (exposure > 400 μ s) and bright (exposure < 100 μ s) regions across frames, indexed 1–84 in columns of 6 tiles each from top left.

We then applied FLI^3^M to a histology slice of human lung tissue, focusing on biologically complex areas. While H&E staining remains the gold standard in histopathology due to its simplicity and ease of interpretation, direct imaging using tissue autofluorescence offers complementary advantages, such as providing intrinsic molecular contrast and revealing distinct endogenous fluorophores [33,34]. However, collecting autofluorescence in lung tissue presents challenges due to low intrinsic fluorescence and the low abundance of natural fluorophores [35]. Imaging unstained tissue thereby often requires longer exposure times (milliseconds) and high light doses (milliwatts), which can lead to inevitable photodamage and prolonged acquisition time [26]. In this experiment, we demonstrated the capability of FLI^3^M to reveal intricate tissue structures that are not resolvable with conventional methods, all within the same runtime, as shown in Fig. 4. The reference H&E image (Fig. 4(a)), scanned by a slide scanner (Zeiss, AxioScan.Z1), shows regions containing an airway (*), inflammatory cell aggregates, tumor nests (**), and—toward the right of the sample—non-cancerous lung tissue (***). The same area, highlighted in black, was imaged on the unstained slide compositing 6 × 14 individual image tiles. Figures 4(b) and 4(d) show the stitched intensity-based images of pre-scan and intensity-inverted, respectively. The pre-scan and intensity-inverted imaging stages were completed in sequence for each tile. The acquisition time of a single pre-scan is 8.4 s and the same for all tiles, followed by the intensity inverted imaging with runtime ranging from 13 s to 43 s, depending on the image content, resulting in a total acquisition time of 2090 s. The co-registered intensity and H&E image (Fig. 4(c)) shows good spatial agreement.

For a fair comparison, a second dataset using raster scan with uniform exposure was collected. The average runtime for the intensity-inverted approach over all tiles was calculated (i.e., the average time of completing the pre-scan and intensity-inverted imaging, 24 s or equivalently 380 μ s in dwell time) and used as the frame time in uniform exposure raster scanning. The raster scan was conducted under the same area and number of tiles, shown in Fig. 4(e). The lifetime results are shown in Fig. 4(f), showing pronounced differences in fluorescence lifetime between cancerous tissue (middle) and alveolar regions (right). The variations in fluorescence lifetime observed are likely due to a combination of biological and environmental factors. Differences in fluorophore composition, including the relative abundance of NADH, FAD, collagen, and elastin, contribute to distinct lifetime signatures, with collagen-rich regions exhibiting longer lifetimes (typically 2–4 ns) and metabolically active areas showing shorter lifetimes [3]. Structural and metabolic heterogeneity between cancerous and non-cancerous tissues further influences fluorescence decay, with altered oxidative states and extracellular matrix remodeling affecting fluorophore interactions [36]. Local environmental factors such as pH fluctuations, oxygenation, and microviscosity may also play a role, modifying excited-state lifetimes through quenching and shifts in emission properties. Optical scattering, tissue density, and hemoglobin absorption introduce additional complexity, altering signal distribution therefore affecting overall lifetime measurement [37]. While this study does not focus on establishing cross-sample statistical significance for the observed lifetime values, the lifetime distinctions between cancerous and non-cancerous tissue observed here are consistent with prior research on lung tissue using autofluorescence [33,38]. To quantitatively determine the lifetime improvements, the lifetime fitting statistics for all tiles are shown in Fig. 4(g). Dim and bright regions are specified based on the exposure adjustments (dim: adapted exposure > 400 μ s, bright: adapted exposure < 100 μ s). Pixels within these regions that have available lifetime data are included in the fitting error calculation, while pixels with missing lifetime data are discarded. Method FLI^3^M on average improves the fitting error by 56% in dim regions. In the bright regions, both approaches show similar performance, reflecting minimal signal loss as the purpose for avoiding unnecessary photodamage.

**Fig. 5.**
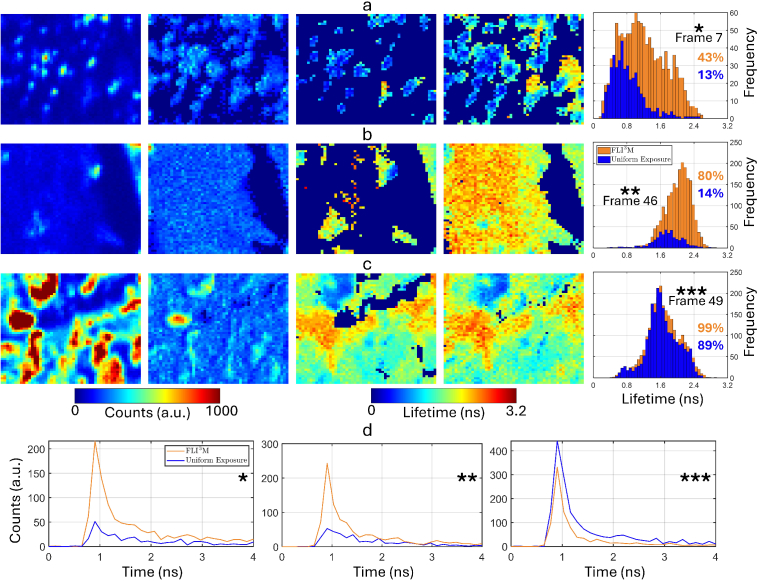
Comparison of intensity and lifetime for three selected areas of interest: **a** Frame 7 (airway region), **b** Frame 46 (mixed tumor/normal tissue), and **c** Frame 49 (multiple distinct species). Intensity and lifetime images without transparency modulation are presented for comparison, including uniform exposure (left) and FLI^3^M (right). Corresponding lifetime histograms are displayed on the far right. The ratio of valid lifetime pixels to the total number of pixels is specified for each method. The photon count distribution is provided in Supplementary Fig. S3 (see Supplement 1). **d** Representative fluorescence decay curves from each marked region, extracted and summed over a 3 × 3 pixel window.

These observations are further supported by the analysis of the contrast between fluorescence lifetimes of different tissues. Typically, lifetime image histograms are used for object-level analysis in regions of interest [39]. In Fig. 5, we selected three of such regions from different tiles to illustrate the improved detection of tissue heterogeneity and structural variations using FLI^3^M. From the lifetime histograms in Figs. 5(a) and 5(b), species with lifetimes exceeding 2 ns are undetectable using the uniform exposure method. This limitation arises from the complex signal characteristics: short-lifetime species appear bright in intensity, whereas longer-lifetime species exhibit weaker fluorescence. In the most challenging region with mixed tumor and normal tissue (Fig. 5(b)), our method offers up to a 66% improvement in lifetime availability. While this level of image quality can be achieved with the conventional approach, it requires over 4,488 seconds of imaging time, making our method 53% faster. Figure 5(c) highlights a region with strong fluorescence, where FLI^3^M employs shorter exposure times. In contrast, the most fluorescence-active areas, depicted in deep red, are overexposed by the uniform exposure method, increasing the risk of photodamage and unnecessarily prolonging the imaging duration. The lifetime histograms for both methods show minimal differences due to high enough SNR. However, FLI^3^M has a slightly broader distribution due to the increased availability of lifetime data in the upper portion of the image. This broader distribution has a negligible impact on the overall shape of the histogram and does not compromise the ability to discern lifetime contrasts. Figure 5(d) shows representative fluorescence decay profiles from the three tissue regions, each exhibiting different decay signatures. While the airway and tumor regions display measurable lifetimes, their decay profiles are poorly resolved under the conventional method—a limitation that is mitigated by FLI^3^M. In the multi-species region, despite lower signal amplitudes, FLI^3^M preserves both the shape and temporal characteristics of the decay curves, capturing the complete optical fingerprints of the tissue. Collectively, the improvements in lifetime availability and reliability, supported by the enhanced statistical strength of the fit, enable robust differentiation between cellular regions with significant variations in both intensity and lifetime.

## 4. Discussion

This study aimed to demonstrate the capabilities of the proposed adaptive imaging method for addressing the challenges of low-SNR imaging without compromising time efficiency. While the system can dynamically adjust exposure times across a high dynamic range and with fine resolution, limitations in scanner hardware currently restrict its ability to achieve video-rate imaging without introducing field distortion—a common issue in laser scanning systems. Image corrections can be applied during post-processing to compensate for distortion in most laser scanning systems [40]. However, such corrections are typically effective only for images acquired with uniform scan patterns, where scanner speed changes are abrupt but symmetric and confined to image boundaries. In contrast, the distortion observed in our system is distance-dependent and occurs throughout the image. To mitigate this, the current system introduces settling pixels between sequential but spatially distant ROI pixels, allowing sufficient time for scanner repositioning. This time redundancy could be reduced by implementing a feedback circuit between the scanner and the AWG, enabling real-time reporting of position errors between commanded and actual voltages. More advanced path-planning strategies, such as nearest-neighbor and minimum spanning tree algorithms [41,42], could further optimize scanning efficiency by finding optimal or near-optimal paths. These approaches, however, must be applied carefully, as they are often computationally expensive and may introduce additional distortion along diagonal directions compared to the unidirectional pathing strategy used in this work. Applying the system to high-frame-rate imaging for *in vivo* FLIM by incorporating real-time feedback mechanisms is the scope of future work. For fluorescence lifetime determination, the CMM estimator assumes a single-exponential decay model. While this approach provides only an averaged lifetime in regions with multiple emitting species, it is often sufficient for diagnostic applications, where lifetime contrast is typically more critical than precise absolute lifetime values [43].

## 5. Conclusion

In summary, this study demonstrates a highly flexible and adaptive fluorescence lifetime imaging system that enables per-pixel exposure control and arbitrary-path scanning. By dynamically adjusting dwell times based on local fluorescence intensity, the system optimizes SNR without compromising imaging speed, effectively addressing key limitations of conventional scanning-based FLIM techniques. Validated on a range of samples, including biologically complex human lung tissue, the system demonstrated a 56% improvement in fluorescence lifetime estimation reliability in low-SNR regions and a 27% to 53% increase in imaging speed across different samples. By dynamically optimizing imaging parameters in response to sample-specific fluorescence variations, this approach represents a significant advancement toward more accurate, efficient, and informative lifetime-based imaging for biomedical research and clinical diagnostics.

## Supplemental information

Supplement 1 supplemental figures/algorithms https://doi.org/10.6084/m9.figshare.29469737

## Data Availability

Data underlying the results presented in this paper are not publicly available at this time but may be obtained from the authors upon reasonable request.

## Supplemental document

See Supplement 1 for supporting content.
