# Supplementary material for: Adaptive fluorescence lifetime imaging with per-pixel signal optimization and flexible scanning
Source: Biomed Opt Express. 2025 Sep 23;16(10):4129–43. doi: 10.1364/BOE.566518 (PMC12532327; doi:10.1364/BOE.566518)
Supplement: Supplementary file 1 [file boe-16-10-4129-s001.pdf]

Supplemental Document

Biomedical Optics EXPRESS

Adaptive fluorescence lifetime imaging with per-pixel signal optimization and flexible scanning: supplement

SIYUAN XIE,[1] GARETH O. S. WILLIAMS,[1] AHSAN R. AKRAM,[1] AHMET T. ERDOGAN,[2] AND JAMES R. HOPGOOD[3,*]

[1]*Centre for Inflammation Research, Institute for Regeneration and Repair, The University of Edinburgh, Edinburgh, UK*
[2]*School of Engineering, Institute for Integrated Micro and Nano Systems, The University of Edinburgh, Edinburgh, UK*
[3]*School of Engineering, Institute for Imaging, Data, and Communications, The University of Edinburgh, Edinburgh, UK*
[*]*James.Hopgood@ed.ac.uk*

This supplement published with Optica Publishing Group on 23 September 2025 by The Authors under the terms of the Creative Commons Attribution 4.0 License in the format provided by the authors and unedited. Further distribution of this work must maintain attribution to the author(s) and the published article's title, journal citation, and DOI.

Supplement DOI: https://doi.org/10.6084/m9.figshare.29469737

Parent Article DOI: https://doi.org/10.1364/BOE.566518

Adaptive fluorescence lifetime imaging with per-pixel signal optimization and flexible scanning: supplement

1. SUPPLEMENTARY ALGORITHMS

A. FLI^{3}M

Algorithm S1. Algorithm for Pixel Classification and Intensity Inversion

Input: Pre-scan intensity image $P(m,n|T_{\text{prescan}})$, sensor dark count rate DCR, intensity target I_{target}, and adaptive exposure time T_{baseline}

Step 1: ROI Map Thresholding

For each image pixel (m,n)

Compute adaptive Otsu's thresholds:

$$O_{\text{thresh}}(m,n) = \text{Otsu}\left(P(m,n \mid T_{\text{prescan}})\right)$$

Compute ROI map thresholds:

$$\alpha(m,n) = \max(\text{DCR}, O_{\text{thresh}}(m,n))$$

EndFor

Step 2: Pixel Classification and Intensity Inversion

For each pixel (m,n)

If $P(m,n|T_{\text{prescan}}) > \alpha(m,n)$

Compute exposure scaling factor:

$$S(m,n) = \frac{I_{\text{target}}}{P(m,n|T_{\text{prescan}})}$$

Compute adaptive exposure:

$$E(m,n) = T_{\text{baseline}} \cdot S(m,n)$$

Else

$E(m,n) = 0$

EndIf

EndFor

Output: Adaptive exposure map $E(m,n)$

Algorithm S2. Algorithm for Coordinate-to-Voltage Mapping

Input: Adaptive exposure map $E(m,n)$, scanner step size V_s, AWG sampling rate f, settling coefficient β
Step 1: Compute voltage sequences
For each pixel (m,n)
    If $E(m,n) \neq 0$
        If $E(m-1,n-1) \neq 0$
            $\vec{V}_x(m,n) = nV_s\vec{1}_{1\times fE(m,n)}$
            $\vec{V}_y(m,n) = mV_s\vec{1}_{1\times fE(m,n)}$
        Else
            Compute transition delay: $\Delta = f + \beta \max(|m - m_1|, |n - n_1|)$
            $\vec{V}_x(m,n) = nV_s\vec{1}_{1\times \Delta E(m,n)}$
            $\vec{V}_y(m,n) = mV_s\vec{1}_{1\times \Delta E(m,n)}$
        EndIf
    Else
        $\vec{V}_x(m,n) = \varnothing$
        $\vec{V}_y(m,n) = \varnothing$
    EndIf
EndFor
Output: Voltage sequences $\vec{V}_x(m,n)$ and $\vec{V}_y(m,n)$

2. SUPPLEMENTARY FIGURES

A. FLI^{3}M

Figure S1 shows the galvanometer scanner response to a step input, used for estimating and compensating scan error. The yellow trace represents the input voltage command, while the blue trace corresponds to the actual scanner position, measured via the built-in position feedback. The step input corresponds to approximately 530 µm of travel on the object plane, equivalent to 300 image pixels. As observed, the scanner requires approximately 600 µs to reach a steady state. The response is approximated as linear during this transition. At a waveform generator sampling rate of 10 MS/s ($f = 10^7$), this results in approximately 20 samples per pixel during settling, giving a settling coefficient of $\beta = 0.02f$.

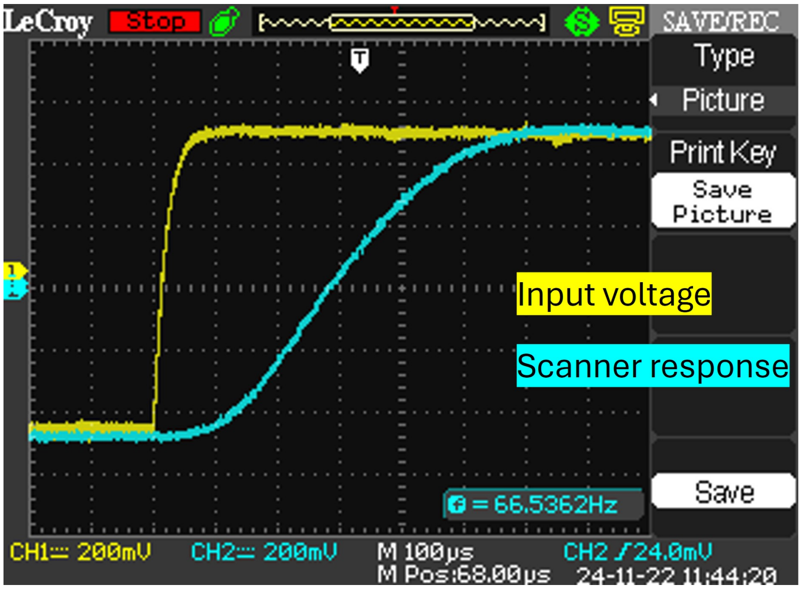

Fig. S1. Galvanometer scanner response to a step input, monitored using an oscilloscope.

B. FLI^{3}M of *Convallaria majalis*

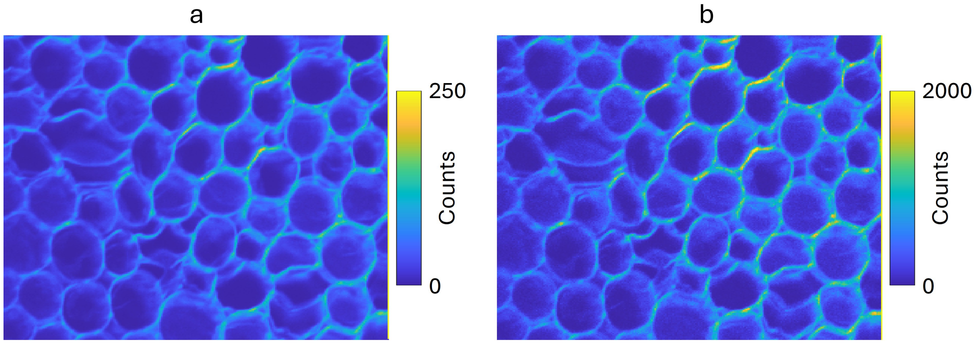

Fig. S2. Pre-scan intensity images from Experiment II **a** and Experiment IV **b**. Both images are time-resolved and were acquired in 7.57 s.

C. FLI^{3}M of unstained human lung tissue for histology

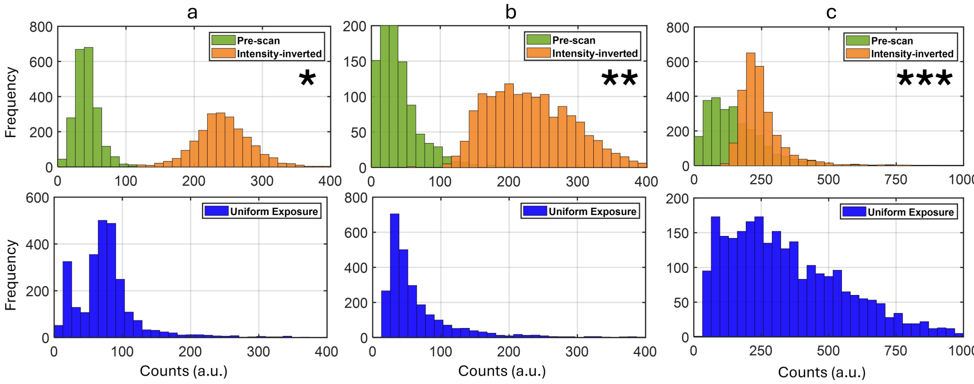

Fig. S3. Comparison of intensity histograms for three highlighted regions: **a** an airway (*), **b** a cancerous region (**), and **c** a structurally complex region (***), shown for FLI^{3}M (top) and uniform exposure (bottom).

D. FLI^{3}M of non-fixed human lung tissue

Figure S4 illustrates the application of FLI^{3}M to fresh, *ex vivo* human lung tissue prior to histological processing. Figure S4b shows the pre-scan intensity image acquired over the region highlighted in Figure S4a. The lifetime image, shown in Fig. S4c, reveals several regions with missing lifetime data due to low photon counts, highlighting the challenges associated with obtaining complete lifetime profiles. A major limitation of imaging unfixed tissue is the absence of structural reference points that are typically available in fixed samples. As a result, intensity variations are difficult to interpret without corresponding lifetime information—they may arise from environmental fluctuations, fluorophore degradation, or incomplete sample coverage. Furthermore, the inherently weak autofluorescence of fresh lung tissue adds complexity to data interpretation. These issues typically become apparent only during post-processing (Fig. S4c), at which point lost information cannot be recovered.

To mitigate this information loss, we used the average intensity of the top 10% brightest pixels per tile in Figure S4b as the target for adaptive imaging. The resulting lifetime image, shown in Fig. S4d, reveals meaningful lifetime contrasts in regions that previously lacked valid data. Notably, the data acquisition for Figure S4d relied solely on the intensity profiles from Figure S4b, providing a fast and efficient method for signal correction. A common strategy to address

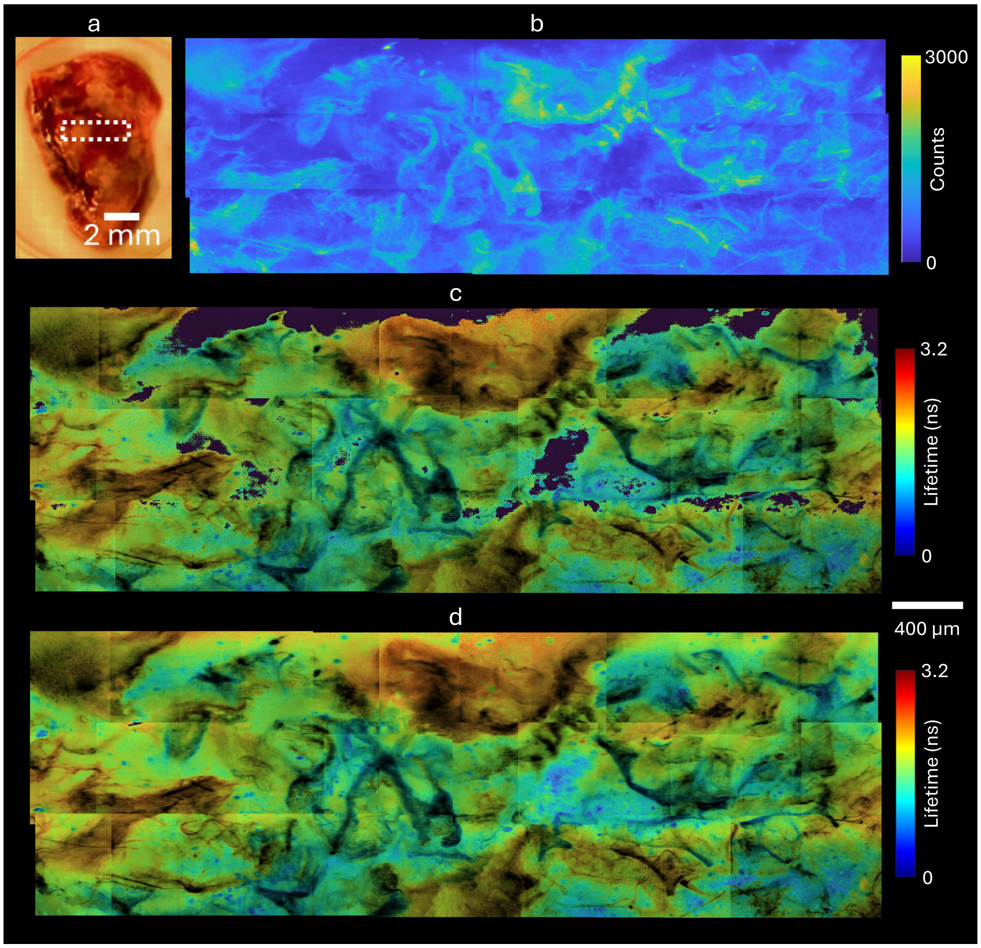

Fig. S4. FLI^{3}M imaging of fresh, *ex vivo* human lung tissue. **a** Macroscopic image of the lung tissue sample during measurement. The dashed box indicates the imaged region, consisting of 3 × 12 stitched images (each 256 × 256 pixels, ∼450 µm × 450 µm), covering a total area of approximately 1.3 mm × 5.4 mm. **b** Pre-scan intensity image of the fresh *ex vivo* tissue, corresponding to the dashed region in panel a. Imaging parameters: 100 µs exposure time, 735 µW excitation power, 475 nm excitation wavelength. **c** Pre-scan lifetime image. The threshold for lifetime estimation was set at five times the dark count level (∼100 photons). **d** Fluorescence lifetime image acquired using sequential intensity-inverted imaging. The target intensity per tile was set to the average photon count of the brightest 10% of pixels within each tile. Adaptive exposure per pixel ranged from 100 µs to 800 µs in 1 µs increments. All image pixels fall within the designated region of interest. Imaging times: 302 s (pre-scan) and 744 s (adaptive imaging). Lifetime images are displayed with inverted transparency to highlight regions with low photon counts.

poor SNR is to globally increase the exposure time. However, without knowledge of optimal excitation conditions, this often leads to inefficient imaging—either overexposing bright regions or underexposing dim ones. Even when similar image quality is achieved, conventional FLIM would require 1,959 seconds, whereas our adaptive method completed acquisition in just 744 seconds, achieving a 46% reduction in imaging time.
